# Austin District Medical Society

**Published:** 1889-01

**Authors:** T. J. Bennett

**Affiliations:** Secretary


### AUSTIN DISTRICT MEDICAL SOCIETY.

### SIXTH QUARTERLY MEETING.

On the 20th of December last the Austin District Medical Society held its sixth quarterly meeting in Austin in three sessions, a forenoon, an afternoon and a night session. It was a very successful meeting, there being thirty-four physicians in attendance, nineteen of whom were from the district outside of the city of Austin. The papers read were a good average and the discussions which followed were interesting and instructive.

The following names were added to the list of membership:

Dr. S. G. Harper, of Hutto;
Dr. J. S. Brown, of Taylor;
Dr. D. Monroe, of Cameron;
Dr. E. T. Gazley, of Austin.

The society is growing and prosperous; there are now fifty-four members, and a number of applications from prominent physicians accessible to Austin lying over for action at the next regular meeting in March.

The following papers were read:

"Management of the Card," by Dr. W. T. Richmond, of Manor.

"Typhlitis," by Dr. T. D. Wooten, of Austin.

"Haematuria," by Dr. O. L. Williams, of Chappell Hill,

"Quinine," by Dr. J. T. Bennett, of Austin.

"Insanity—Its Cause and Prevention," by Dr. A. N. Denton, of Austin.

"Medical Legislation," by Dr. R, P. Talley, of Belton.

Dr. Talley's paper was read at the night session, but was not received, and therefore not discussed. It was an attempted argument against medical legislation, but was in fact, no argument. It consisted mainly of personalities, ridicule and abuse of all former attempts at legislation by the organized profession.

Dr. R. S. Gregg, of Manor, offered the following resolution, which is to become an amendment to the by-laws:

*Resolved:* That no member shall be allowed to occupy more than twenty minutes in reading a paper, nor occupy the floor longer than ten minutes in discussion, unless the Society by a majority vote grant a longer time.

T. J. BENNETT, Secretary.

